# A Case Series of Problems That Can Occur During Open Heart Surgery in Patients With COVID-19 Infection

**DOI:** 10.7759/cureus.27488

**Published:** 2022-07-30

**Authors:** Senem Girgin, Murat Aksun, Ahmet Salih Tüzen, Ali Gürbüz, Nagihan Karahan

**Affiliations:** 1 Department of Anesthesiology and Reanimation, Izmir Katip Celebi University Atatürk Training and Research Hospital, Izmir, TUR; 2 Department of Cardiovascular Surgery, Izmir Katip Celebi University Atatürk Training and Research Hospital, Izmir, TUR

**Keywords:** covid-19 infection, coronavirus disease 2019, open heart surgery, post-operative complications, peri-operative outcomes, extracorporeal circulation, covid-19

## Abstract

The utilization of open cardiac surgery on patients infected with coronavirus disease 2019 (COVID-19) has resulted in a very challenging perioperative management method. High rates of morbidity and mortality have been documented in the literature for patients who have undergone open heart surgery while infected with COVID-19; however, data on complications that may occur during and after surgery in patients with COVID-19 infection are limited. In this article, we aimed to present the clinical course and perioperative consequences of three patients with preoperative COVID-19 infection.

## Introduction

Open heart surgery in patients with coronavirus disease 2019 (COVID-19) infection creates various problems, such as coagulopathy, hypoxia, difficulty weaning, and susceptibility to secondary bacterial infections. In addition to surgical trauma, impaired extracorporeal circulation and impaired myocardial and pulmonary reserve can lead to immunosuppression and coagulopathy, which further complicate the management of these patients [1,2].

Infection with COVID-19 can result in serious cardiovascular consequences, including direct myocardial injury, severe systemic inflammatory response, hypoxia, coronary thrombosis, and plaque rupture. There are increased risks of postoperative complications for surgery in a patient with previous COVID-19 infection, such as pulmonary dysfunction, arterial embolism, venous thrombus, cardiac dysfunction, residual neuromuscular weakness, and difficulty weaning [3,4].

In this article, we aimed to present the clinical course and perioperative complications of three patients who had preoperative COVID-19 infection in the cardiac surgery process.

## Case presentation

Case 1

**Figure 1:**
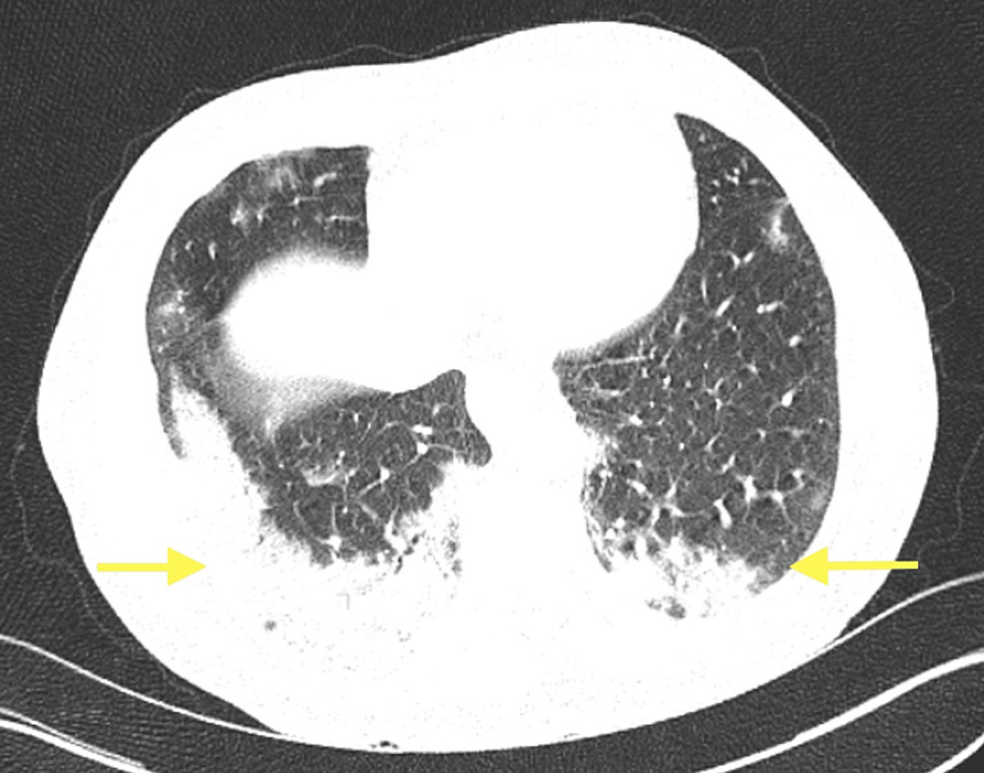
Pneumonic infiltrates in the bilateral basal zone in thorax CT

**Figure 2:**
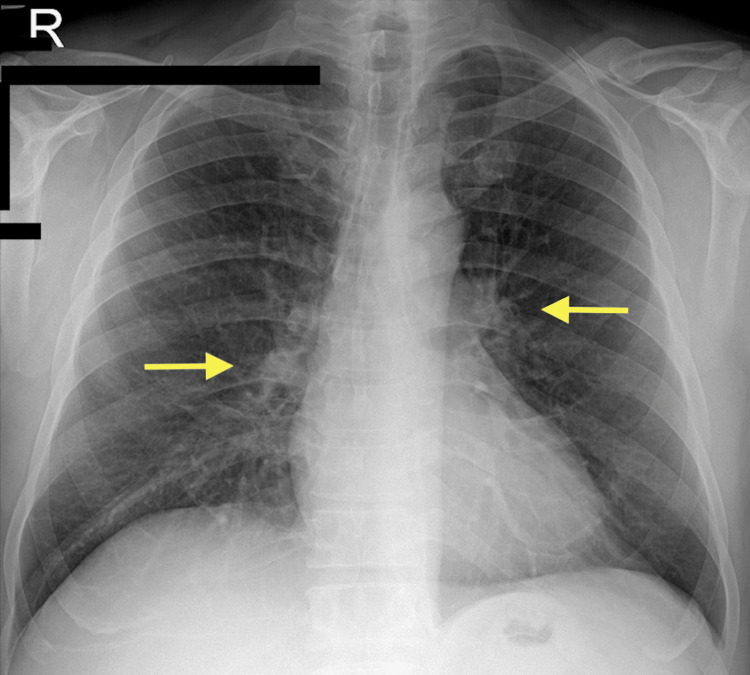
Hilar bronchiectasis (arrows) but no active infiltration on preoperative chest X-ray

A 63-year-old male patient, without any significant past medical history, was diagnosed with COVID-19 infection three months ago and admitted to the hospital when he developed dyspnea during his home treatment. His chest computed tomography (CT) revealed pneumonia, and he was discharged after 11 days of hospitalization (Figure 1). Three months later, the patient presented with chest pain to the cardiology outpatient clinic. Transthoracic echocardiography (TTE) revealed hypokinesia in the posterobasal left ventricle. TTE showed a left ventricular diastolic diameter of 53 mm and a left ventricular systolic diameter of 38 mm, with an ejection fraction (EF) of 50%. Bronchiectasis was present in the hilar region on the preoperative chest X-ray, but no active infiltration was observed (Figure 2). Hemogram, biochemistry, and coagulation parameters were normal, and a negative polymerase chain reaction (PCR) was detected prior to surgery. After coronary angiography revealed multiple and severe coronary artery stenosis, cardiopulmonary bypass (CPB) and coronary artery bypass grafting (CABG) were routinely performed. Perioperative blood gas values and hemodynamics were normal. The patient underwent acute normovolemic hemodilution and did not receive a blood or blood product transfusion. After 600 ml of chest tube drainage in the fourth postoperative hour, he was transported for revision surgery due to extensive bleeding. No surgical bleeding site was identified. The patient had thrombocytopenia and was transfused with six units of random platelets. The platelet count before transfusion was 43 x 10^9^/L and the platelet count after the transfusion was 82 x 10^9^/L. The patient also exhibited a prolonged weaning period of 18 hours.

Case 2

**Figure 3:**
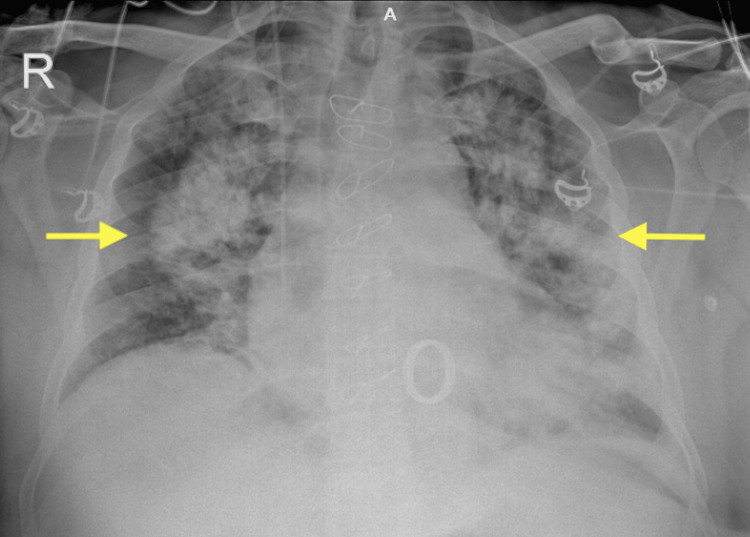
Multilobular bilateral pulmonary infiltration on chest X-ray

A 47-year-old male patient with a past medical history of obstructive sleep apnea was admitted to the COVID-19 department due to respiratory distress while he was scheduled for elective mitral valve replacement (MVR) due to severe mitral regurgitation. After two days of oxygen mask therapy, he was discharged from the hospital. After three months, he presented for elective MVR at our clinic. Both the pulmonary function test and the preoperative chest CT were normal. TTE revealed left atrial enlargement in addition to mitral regurgitation of the third degree. TTE revealed the following findings: left ventricular diastolic diameter of 47 mm, left ventricular systolic diameter of 22 mm, left atrial diameter of 40 mm, EF of 60%, third-degree mitral regurgitation, mitral valve prolapse to the left atrium, rupture of mitral posterior cord, and elongated mitral anterior chord. MVR was performed utilizing the standard intraoperative CPB technique. Postoperative arterial blood gas levels were hypoxic. He was extubated at postoperative hour eight, but on postoperative day one, he developed fever, dyspnea, and new consolidations in the chest X-ray consistent with pneumonia (Figure 3). Laboratory findings of the patient included high procalcitonin level, elevated C-reactive protein, and leukocytosis. No growth was observed in postoperative culture specimens and COVID-19 PCR was negative. Antimicrobials were administered. It was considered a secondary bacterial infection.

Case 3

**Figure 4:**
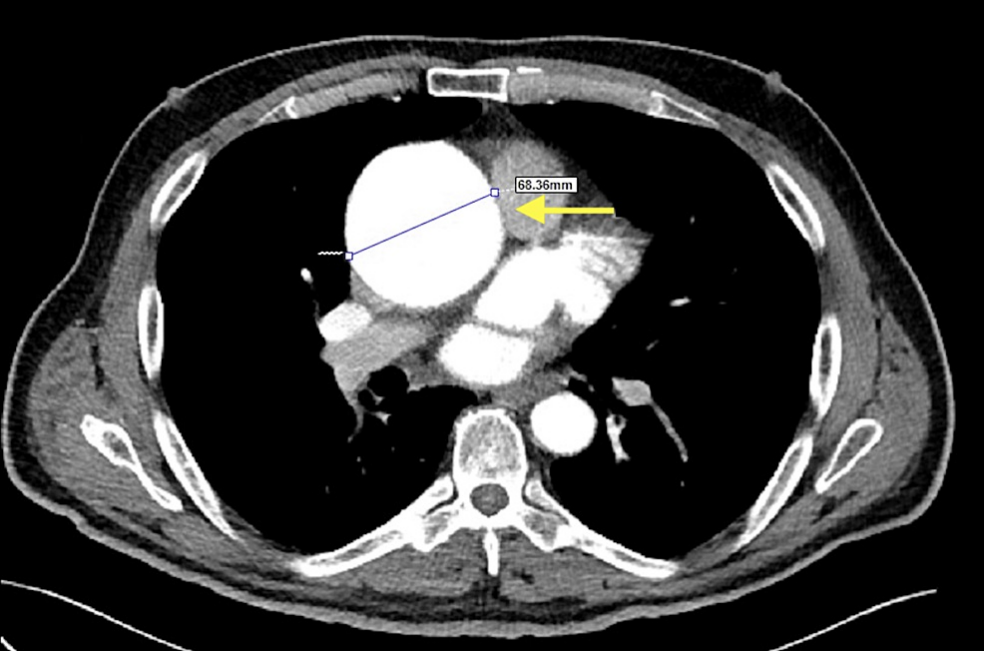
Ascending aortic aneurysm and transverse diameter of 69 mm on CT angiography

**Figure 5:**
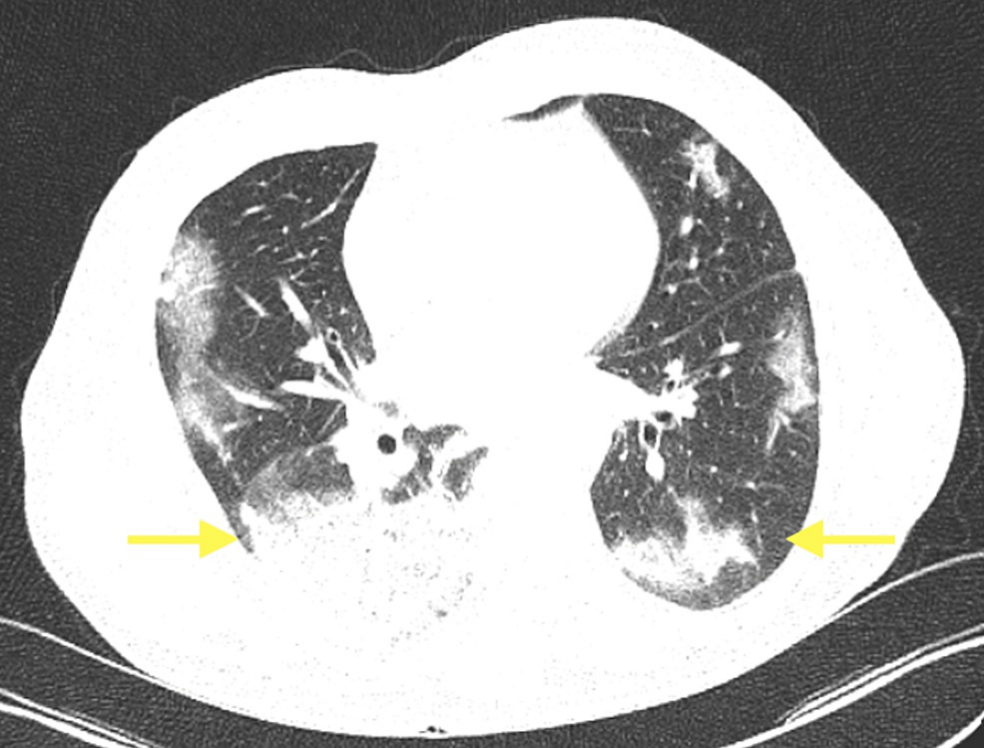
Interlobular septal thickening and ground glass with unclear border on CT thorax

A 60-year-old male patient presented to the cardiology outpatient clinic with a complaint of atypical chest pain. TTE revealed the following findings: left ventricular diastolic diameter of 68 mm, left ventricular systolic diameter of 54 mm, left atrial diameter of 43 mm, ascending aorta transverse diameter of 69 mm, EF of 35%, global hypokinetic left ventricular wall movements, and severe aortic regurgitation. On CT angiography, the diameter of the ascending aorta was measured to be 68.3 mm (Figure 4). His electrocardiogram showed a normal sinus rhythm. The council decided on early aortic ascending surgery. Preoperatively, a positive PCR was noted. Chest CT showed emphysematous, interlobular septal thickening in inferior zones, and ground glass with unclear borders; the appearance favored volume strain in the interstitium (Figure 5). The pulmonary function test was moderately restrictive. After a five-day follow-up in the COVID-19 service, PCR negativity was noted twice (two weeks after a positive PCR) and Bentall surgery (aortic root and aortic valve replacement) was performed. No complication was observed except prolonged weaning (16 hours).

Surgical perioperative information, including EuroSCORE II, cross-clamp time, type of surgery, CPB time, extubation time, length of intensive care unit stay, and length of hospital stay, is shown in Table 1.

**Table 1:** Surgical perioperative data of the cases CPB: cardiopulmonary bypass; CABG: coronary artery bypass grafting; MVR: mitral valve replacement.

	Age	Sex	Operation	Cross-clamp time (minute)	CPB time (minute)	Extubation time (hour)	Length of ICU stay (day)	Length of hospital stay (day)	EuroSCORE II
Case 1	63	Male	CABG	43	83	18	3	8	2.13
Case 2	47	Male	MVR	100	133	8	6	13	1.36
Case 3	60	Male	Bentall	82	135	16	3	10	12.65

## Discussion

The World Health Organization (WHO) declared COVID-19 a pandemic in March of 2020, causing extremely severe health issues worldwide. Currently, our experience in treating emergency and elective patients who have undergone open heart surgery is expanding, and unfortunately, high rates of morbidity and mortality have been reported in patients who underwent cardiac surgery while infected with COVID-19, regardless of age or pre-existing conditions [1]. Today, an increasing number of patients who have recovered from the infection, which will have an effect on our clinical practice, are presenting for elective and emergency procedures in hospitals. Major elective surgery performed between zero and four weeks after COVID-19 infections are linked with an increased incidence of postoperative complications; surgery performed between four and eight weeks later is linked with an increased incidence of postoperative pneumonia. It is seen that COVID-19-positive patients who have undergone heart surgery stay longer in hospital after surgery, stay longer in the intensive care unit, have high morbidity and mortality, and have poor outcomes [3]. In addition to causing surgical trauma, cardiopulmonary bypass results in a severe systemic inflammatory response syndrome. CABG and surgical trauma lead to a transient immunosuppressive state in severity and duration [5]. In addition, SARS-CoV-2 leads to a proinflammatory and procoagulant state causing endothelial and microvascular dysfunction. High levels of D-dimer and fibrinogen are significantly associated with hospitalization, disease severity, and mortality [6].

Possible pathophysiological mechanisms leading to coagulopathy during active COVID-19 infection are currently under investigation [7]. However, the long-term coagulation issues of COVID-19-infected patients are unclear. About 60% of patients hospitalized with COVID-19 infection develop coagulopathy such as thrombocytopenia, hypercoagulation, disseminated intravascular coagulation (DIC), and venous thrombosis [8]. The occurrence of postoperative drainage in the third case prompts us to believe that we require additional information regarding the effects of extracorporeal circulation on the coagulation cascade. There appears to be an increase in the frequency of cardiac arrhythmias and sudden cardiac arrest among COVID-19-infected hospitalized patients. These pathologies are thought to be the result of heart damage, specifically due to hypoxia, deteriorating coronary perfusion, sepsis, direct myocardial damage, and the proarrhythmic effect of a few COVID-19 treatment drugs. The pro-inflammatory state caused by the disease with COVID-19 also plays an important role in this process [7,8].

In addition to cardiopulmonary bypass, invasive mechanical ventilation and ischemia-reperfusion injury contribute significantly to this process. However, COVID-19 disease-related myocardial damage may also be a significant factor [9]. A study of patients with COVID-19 admitted to the intensive care unit revealed that one-third of patients had new-onset cardiomyopathy with clinical signs of cardiogenic shock, including decreased left ventricular work and elevated cardiac proteins. In addition, 40% of COVID-19 fatalities were accompanied by concomitant heart failure [10-12]. The high cardiometabolic burden of systemic inflammation is associated with myocardial damage resulting from prolonged hypoxia due to pneumonia or acute respiratory distress syndrome [13]. This process can also result in plaque rupture and acute coronary syndrome [14].

Complications such as coagulopathy, secondary bacterial infections, and prolonged weaning, which occurred in these three cases, are common in open heart surgery and may have many causes. Nonetheless, there are few studies in the current scientific literature that demonstrate the association between these complications and previous COVID-19 infection with multisystem involvement [15]. COVID-19 patients' perioperative mortality (24.5% vs. 24.8%) is comparable to general mortality data (24%) and specialties reporting from multiple surgeries (23.8%) from the COVIDSurg Collaborative [16]. Unpostponed emergencies and emergency surgery are likely to have poorer postoperative results [17,18]. In times of the COVID-19 pandemic, the optimal timing of cardiovascular surgery has become even more delicate, as the morbidity associated with the disease increases due to delays [19].

In a study examining respiratory parameters after cardiac surgery in asymptomatic COVID-19 patients, the rate of readmissions to the intensive care unit was higher in COVID-19 patients than in the group without COVID-19, but the data on respiratory complications that may develop in COVID-19 patients postoperatively are limited [20]. We had complications such as secondary bacterial pneumonia in case 2 and prolonged weaning time in case 1 and case 3 (Table 1).

We would like to emphasize the urgent need to develop defined protocols based on evidence for the preoperative assessment and intraoperative and postoperative management of these patients who had COVID-19 infection prior to open heart surgery.

## Conclusions

Increasing numbers of patients with COVID-19 undergo cardiac surgery. It is essential to understand the post-COVID-19 effects to determine the optimal timing of surgery and the morbidity associated with surgery. In patients with COVID-19, cardiovascular surgery may soon encounter a new challenge, especially in light of the knowledge of cardiac effects and cardiac complications gained in the subsequent time period. In addition to the preoperative and intraoperative phases, postoperative pulmonary and cardiac complications must be carefully monitored in this patient population.
